# Molecular analyses on host-seeking black flies (Diptera: Simuliidae) reveal a diverse assemblage of *Leucocytozoon* (Apicomplexa: Haemospororida) parasites in an alpine ecosystem

**DOI:** 10.1186/s13071-015-0952-9

**Published:** 2015-06-25

**Authors:** Courtney C. Murdock, Peter H. Adler, Jared Frank, Susan L. Perkins

**Affiliations:** Department of Infectious Diseases, College of Veterinary Medicine, Odum School of Ecology, University of Georgia, Athens, GA 30602 USA; Entomology Program, Clemson University, Clemson, SC 29634-0310 USA; Sackler Institute for Comparative Genomics, American Museum of Natural History, Central Park West at 79th Street, New York, NY 10024 USA

**Keywords:** Haemosporidian, *Leucocytozoon*, Simulium, *cytb* haplotypes, Black fly, Parasite, Malaria

## Abstract

**Background:**

Molecular studies have suggested that the true diversity of *Leucocytozoon* (Apicomplexa: Haemospororida) species well exceeds the approximately 35 currently described taxa. Further, the degree of host-specificity may vary substantially among lineages. Parasite distribution can be influenced by the ability of the parasite to infect a host, vector preferences for certain avian hosts, or other factors such as microhabitat requirements that increase the probability that vertebrate hosts and vectors are in frequent contact with each other. Whereas most studies of haemosporidians have focused on passerine hosts, sampling vectors in the same habitats may allow the detection of other lineages affecting other hosts.

**Methods:**

We sampled abundant, ornithophilic black flies (Simuliidae) across a variety of sites and habitats in the Colorado Rocky Mountains throughout the summer of 2007. Black flies were screened with PCR using *Leucocytozoon*-specific primers that amplify a portion of the cytochrome *b* gene, and the sequences were compared to the haplotypes in the MalAvi database. Infections of *Leucocytozoon* from birds sampled in the same area were also included.

**Results:**

We recovered 33 unique haplotypes from the black flies in this study area, which represented a large phylogenetic diversity of *Leucocytozoon* parasites. However, there were no clear patterns of avian host species or geography for the distribution of *Leucocytozoon* haplotypes in the phylogeny.

**Conclusions:**

Sampling host-seeking vectors is a useful way to obtain a wide variety of avian haemosporidian haplotypes from a given area and may prove useful for understanding the global patterns of host, parasite, and vector associations of these ubiquitous and diverse parasites.

**Electronic supplementary material:**

The online version of this article (doi:10.1186/s13071-015-0952-9) contains supplementary material, which is available to authorized users.

## Background

Members of the genus *Leucocytozoon* are globally distributed avian haemosporidian parasites [1, 2]. These parasites are transmitted among avian hosts by black fly vectors (Diptera: Simuliidae). Although at least 35 morphologically defined species have been described to date [1], molecular studies suggest that the true diversity well exceeds this number e.g. [2–4]. Several of the molecular lineages have demonstrated that the degree of host-specificity varies; some lineages are found consistently only in one bird species, whereas other lineages develop successfully in a wide range of taxonomically varied hosts [2, 3]. Further, species of *Leucocytozoon* can complete development across a large range of simuliid vectors [1, 5]. Because both the vertebrate host and dipteran vector are essential for the complete development of *Leucocytozoon* species [1], the distribution of parasites will be influenced by the ability of the parasite to infect a particular vertebrate host, active vector preferences for certain avian hosts, and other factors such as climatic variables and microhabitat requirements that place vertebrate hosts and vectors in frequent contact with each other [6].

The objective of this study was to sample a set of host-seeking black flies across a variety of sites and habitats in an alpine ecosystem and to use molecular methods to assess the diversity of *Leucocytozoon* parasites in these vectors. Our hypotheses were that the flies would contain a wider variety of parasite lineages than would be obtained by sampling the avian community, and parasite distributions would be structured by avian host or habitat type.

## Methods

### Field site description and study design

Black flies were collected from two field sites within the vicinity of the Rocky Mountain Biological Laboratory (RMBL) in Gothic, Gunnison County, Colorado, U.S.A between May 19 and July 31, 2007. One field site, East River Valley, was located approximately 2 km south from RMBL (UTM: N 4312713 E 327700) and the other field site was located in the adjacent, Washington Gulch valley (UTM: N 4311531 E 325807). Elevation ranges from 2902 m to 2987 m asl. Both field sites were a mosaic of alpine meadows, forest stands, and riparian willow thickets. Meadow habitats consisted of a diversity of herbaceous vegetation across a range of elevations. Forest habitats were composed of conifer (*Picea engelmannii*) and aspen (*Populus tremuloides*) stands at higher elevations. Willow thickets were dominated by bog birch (*Betula glandulosa*), mountain alder (*Alnus tenuifolia*), and several species of willow (*Salix* spp.). To ensure a representative sample of the black fly communities, each field site was stratified by the following habitat types: willow, meadow, and forest. We took a random sample of habitat patches from each field site to ensure we sampled across two patches of each habitat type per field site. The following permits were required for this research: Federal Banding Permit, 2328; Colorado Fish and Wildlife Service License, 09TRb1094; and University of Michigan Animal Care Committee Permit, #9077.

### Black fly collection methods

During the summer of 2007, we captured host-seeking black flies with carbon dioxide-baited Centers for Disease Control (CDC) miniature light traps (John W. Hock Company, No. 512 fine mesh collection cups, Gainesville, FL, USA) (Service, 1976). CDC traps were set and checked every 24 h. Due to the possibility of trap failure, traps were paired approximately 50 m from each other within each sampled patch. We trapped each habitat patch for two consecutive nights over an interval of eight days. After each two-day trapping session, traps were pulled and rotated to the other field site to minimize any potential effects of season or weather on trap success. Sampling occurred May 19–May 26, June 8–June 17, July 1–July 12, and July 22–July 31 in 2007.

To immobilize captured dipterans, we placed them in a bag and exposed them to cotton soaked in triethylamine (Fisher Scientific, Amber Glass, No. BP616-500, Pittsburgh, PA, USA) for five minutes in a well-ventilated area. We then separated the black flies from other biting dipterans and stored them in 95 % ethanol for future identification and parasite DNA analyses. Female black flies were identified to species or species complex based on structural characters [7]. Identifications were facilitated by genitalia preparations of selected specimens. The females of *Simulium silvestre* and *Simulium craigi* are not reliably distinguished by morphological criteria, but for the purposes of this paper, we will refer to them simply as *S. silvestre*. Likewise, *Simulium exulatum* and *Simulium pilosum* are not easily distinguishable and those flies will be referred to as *S. exulatum*. Representative specimens have been deposited in the Clemson University Arthropod Collection, Clemson, South Carolina.

### DNA extraction, amplification, and sequencing methods

Black flies were separated into vials corresponding to morphological identification and sampling date and location. Because of the large number of flies in some traps, we opted to extract DNA from pools of five flies for downstream molecular screening. Each black fly was minced with sterile razorblades. Total DNA was extracted using the DNeasy Animal Tissue extraction kit QIAGEN (Valencia, CA, USA), eluting in 200 μl total of Buffer AE. To screen the black fly samples for *Leucocytozoon* parasites, we conducted a polymerase chain reaction (PCR) on the extracted DNA, using the nested primer set developed by Waldenström *et al.* [8], which amplifies a 479 bp fragment of the mitochondrial cytochrome *b* gene with outer primers that are general to the three common genera of avian haemosporidians and nested primers that are specific to *Leucocytozoon*. PCRs were conducted in 25 μl reactions using 10 μl of TopTaq MasterMix (QIAGEN, Valencia, CA, USA), 1 mM of each primer and 2 μl of DNA extracted from the pooled black fly samples. PCR conditions followed recommended protocols by Waldenström *et al.* [8]. To determine if our sample of black flies had recently fed, and to describe any potential vector feeding patterns, we used vertebrate primers L14816 and H15173 [9] to amplify any vertebrate DNA present in blood meals [10]. Negative and positive controls were always included in PCR reactions to detect possible contamination. Amplified DNA was visualized on a 1.5 % agarose gel with CyberSafe (10 %; Invitrogen, Carlsbad, CA; 2 μl per 100 μl of gel), and positive amplifications were cleaned with the AMPure reagent (Agencourt, Beverly, Massachusetts) and sequenced in both directions using BigDye v. 3.1 (Applied Biosystems, Foster City, California) with the same primers that were used in amplification. Sequences were cleaned with CleanSeq (Agencourt, Beverly, Massachusetts) and run on an ABI 3730×l automated sequencer. We re-amplified and re-sequenced any samples that revealed ambiguous or poor-quality base calls. Black fly pools that showed evidence of multiple infections after sequencing (via multiple peaks in electropherograms) were re-amplified and PCR products were cloned using the TOPO® TA cloning kit for sequencing (Invitrogen, Carlsbad, CA), to separate products. To avoid erroneously incorporating chimeric sequences into our phylogenetic analyses, only sequences from these cloned products that showed base calls matching those in the corresponding directly sequenced products were analyzed.

### Sequence analysis and phylogenetic analyses

**Fig. 1:**
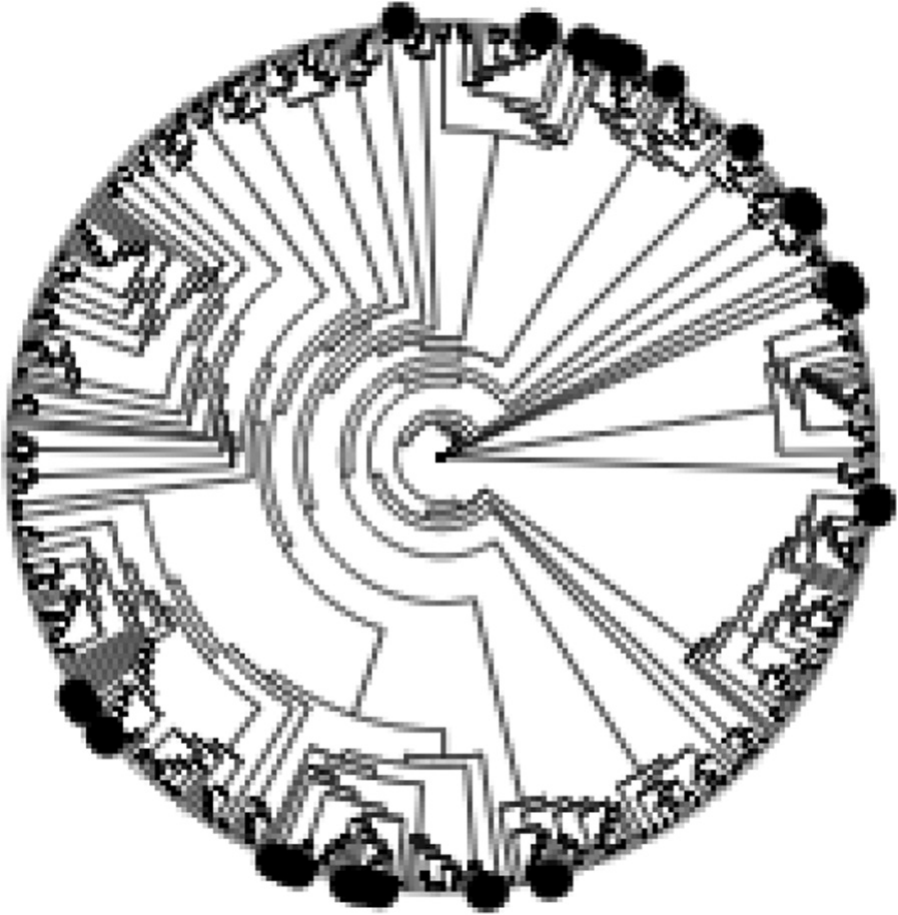
Maximum likelihood phylogeny of *cox1* sequences of *Leucocytozoon* parasites amplified from Colorado black fly pools in the context of all *Leucocytozoon* haplotypes in the MalAvi database. The phylogeny is rooted with five sequences of *Haemoproteus* (*Parahaemoproteus*). Samples from the Colorado flies are indicated with large black dots

Raw sequences were assembled into contigs and edited in Geneious v 6.1.3 (http://www.geneious.com) [11]. The clean sequences generated were incorporated into two phylogenetic analyses. First, all *Leucocytozoon* sequences that were available in the MalAvi database [12] were downloaded on January 30, 2015 (a total of 500 sequences) along with 5 sequences from *Haemoproteus* (*Parahaemoproteus*) species to serve as an outgroup. Sequences were aligned in Geneious using the Geneious Alignment algorithm and default parameters. Although the resulting alignment contained several single-base-pair gaps, likely to be the result of sequencing errors in the deposited sequences, we did not manually edit these gaps in an effort to not introduce additional misalignments in the analysis. The first phylogeny was produced using the RaxML GUI interface [13], with nodal support assessed using the software’s rapid bootstrap algorithm. Following this large analysis, we created a smaller set of MalAvi sequences representing the top BLAST hits for each *Leucocytozoon* haplotype recovered from our black fly samples (Table 1). This reduced data set included any haplotype that had been associated with a named *Leucocytozoon* species in the MalAvi database. Also included in this data set, were nine sequences isolated from seven different passerine hosts. These sequences were generated with the same primers and conditions as for sampled black flies, and came from birds that were sampled across the same field sites and visually or molecularly identified as having single-lineage infections [14]. Nodal support was obtained by using the thorough bootstrap search strategy in RAxML. The phylogeny in Fig. 1 was plotted in RStudio version 0.98.507 using the “ape” package [15].

**Table 1:** Best MalAvi database BLAST hits for *Leucocytozoon* haplotypes found in Colorado black flies

Haplotype	Detected in:	Top BLAST hit	E value
COLBF01	*G. denaria* 9 Jun (2-W)	GALLUS08	5E-152
	*S. silvestre* 26 Jul (2-W)		
	*S. silvestre* 28 Jul (2-F)		
	*S. silvestre* 31 Jul (1-F)		
	*S. silvestre* 31 Jul (1-M)		
COLBF02	*S. exulatum* 14 Jun (2-F)	STOCC13	0.0
COLBF03	*S. silvestre* 30 Jun (1-W)	PARUS25	0.0
COLBF04	*S. silvestre* 30 Jun (1-W)	PARUS25	0.0
COLBF05	*S. silvestre* 2 Jul (1-W)	CB1	0.0
	*S. silvestre* 9 Jul (1-M)		
	*S. silvestre* 10 Jul (1-M)		
	*S. silvestre* 11 Jul (2-M)		
	*S. silvestre* 19 Jul (1-F)		
	*S. silvestre* 19 Jul (1-F)		
	*S. silvestre* 28 Jul (2-F)		
	*S. silvestre* 30 Jul (1-F)		
	*S. silvestre* 30 Jul (1-W)		
	*S. silvestre* 31 Jul (1-M)		
COLBF06	*S. silvestre* 2 Jul (1-W)	EUSE2	0.0
	*S. silvestre* 9 Jul (1-M)		
COLBF07	*S. silvestre* 6 Jul (2-W)	TABI09	0.0
COLBF08	*S. silvestre* 7 Jul (2-M)	TUFAL01	0.0
COLBF09	*S. silvestre* 7 Jul (2-W)	GALLUS08	2E-150
COLBF10	*S. silvestre* 7 Jul (2-W)	TUMER02	0.0
	*S. silvestre* 9 Jul (1-M)		
	*S. silvestre* 11 Jul (2-M)		
	*S. silvestre* 12 Jul (2-W)		
	*S. silvestre* 24 Jul (2-M)		
	*S. silvestre* 29 Jul (2-W)		
COLBF11	*S. silvestre* 9 Jul (1-M)	CNEORN01	0.0
COLBF12	*S. silvestre* 9 Jul (2-M)	CB1	0.0
	*S. silvestre* 19 Jul (1-F)		
COLBF13	*S. silvestre* 10 Jul (1-W)	ZOLEU02	0.0
COLBF14	*S. silvestre* 10 Jul (1-W)	GALLUS08	2E-150
COLBF15	*S. silvestre* 10 Jul (1-M)	TABI09	0.0
COLBF16	*S. silvestre* 11 Jul (2-M)	PARUS25	0.0
	*S. silvestre* 28 Jul (2-W)		
	*S. silvestre* 30 Jul (1-F)		
COLBF17	*S. silvestre* 18 Jul (1-F)	TRPIP2	0.0
COLBF18	*S. silvestre* 18 Jul (1-F)	EMPALN01	0.0
	*S. silvestre* 31 Jul (1-M)		
COLBF19	*S. silvestre* 19 Jul (1-F)	TRPIP2	0.0
COLBF20	*S. silvestre* 24 Jul (1-F)	BUVIR03	0.0
COLBF21	*S. silvestre* 24 Jul (1-F)	EMPALN01	0.0
COLBF22	*S. silvestre* 24 Jul (1-F)	HGR1	0.0
COLBF23	*S. silvestre* 24 Jul (2-M)	TROEAD09	0.0
	*S. silvestre* 25 Jul (1-M)		
	*S. silvestre* 30 Jul (1-W)		
COLBF24	*S. silvestre* 25 Jul (1-M)	GALLUS08	1E-152
	*S. silvestre* 28 Jul (2-F)		
COLBF25	*S. silvestre* 27 Jul (2-W)	CB1	0.0
COLBF26	*S. silvestre* 28 Jul (2-F)	CARFLA04	0.0
COLBF27	*S. silvestre* 29 Jul (2-W)	BUVIR03	0.0
	*S. silvestre* 31 Jul (1-F)		
COLBF28	*S. silvestre* 30 Jul (1-M)	STUR1	0.0
COLBF29	*S. silvestre* 30 Jul (1-F)	EUSE1	0.0
COLBF30	*S. silvestre* 30 Jul (1-F)	EUSE1	0.0
COLBF31	*S. silvestre* 30 Jul (1-W)	TRPIP2	0.0
COLBF32	*S. silvestre* 31 Jul (1-W)	STUR1	0.0
COLBF33	*S. silvestre* 31 Jul (1-F)	HYPHI28	0.0

Sample designation include species of blackfly, date collected in 2007, site (1 = East River Valley, 2 = Washington Gulch), and habitat type (W = willow, F = forest, M = meadow)

## Results and discussion

### Parasite prevalence and blood meal analysis

During the summer of 2007, we collected a total of 2921 black flies including 1929 ornithophilic species specimens belonging to six species/species complexes (Additional file 1). We subsampled from three of five genera and screened a total of 174 pools of black flies with PCR. We detected *Leucocytozoon cytb* amplicons from 67 out of 145 (46.2 %) pools of *S. silvestre*, two out of 14 (14.3 %) pools of *Greniera denaria*, one of the two *Simulium exulatum* pools and no positives from any of the 13 mammalophilic *Prosimulium* spp. pools. Twenty-one of the amplicons from the *S. silvestre* pools and one of the *G. denaria* pools yielded sequences with multiple peaks in the electropherograms. PCR products from these pools were subsequently cloned.

The majority of black flies sampled for *Leucocytozoon* parasites had not fed recently; no sampled black flies had visible blood in their abdomens and only four of the *S. silvestre* pools that were screened with vertebrate-specific primers showed any amplification. These results suggest that parasite amplification could reflect either of the following two scenarios: 1) established and potentially infectious parasite stages rather than parasites present in the blood meal that would not be transmitted, or 2) persisting parasite DNA from abortive sporogony [16]. Of the pools positive for vertebrate DNA, three (SS46d, SS42a, and SS39E) yielded sequences identified as Dusky Grouse (*Dendragapus obscurus*). The fourth pool (SS44A) yielded a sequence identified as an American Crow (*Corvis brachyrhynchos*).

### Diversity of *Leucocytozoon* sequences in Colorado black flies

We obtained 33 unique *cytb* sequences from 60 of the 70 positive black fly pools that were successfully sequenced (Table 1, GenBank Accession Numbers KR052933-KR052965). Twenty-three of the 33 haplotypes were observed only from a single trapping event; however, the other 10 haplotypes occurred in more than one, with several haplotypes detected in different sites, in different habitat types (meadow, forest, or willow) and at different dates throughout the sampling period. Most notably, haplotype COLBF01 was detected in both *S. silvestre* and *G. denaria* and was present in the system from the first sampling period until the last sampling event. Figure 1 presents the full phylogeny of these sequences in the context of the MalAvi *Leucocytozoon* haplotypes from around the globe and from 13 different host orders, rooted with five *Haemoproteus* (*Parahaemoproteus*) sequences. The tips of this tree corresponding to sequences observed in the Colorado black flies are shown with large dots. Although we did not observe Colorado black fly-borne *Leucocytozoon* haplotypes in each of the major clades of the full *Leucocytozoon* tree, Fig. 1 demonstrates that there is a great diversity of *Leucocytozoon* parasites present in this particular vector community, with no evidence that limited *in situ* diversification has occurred.

**Fig. 2:**
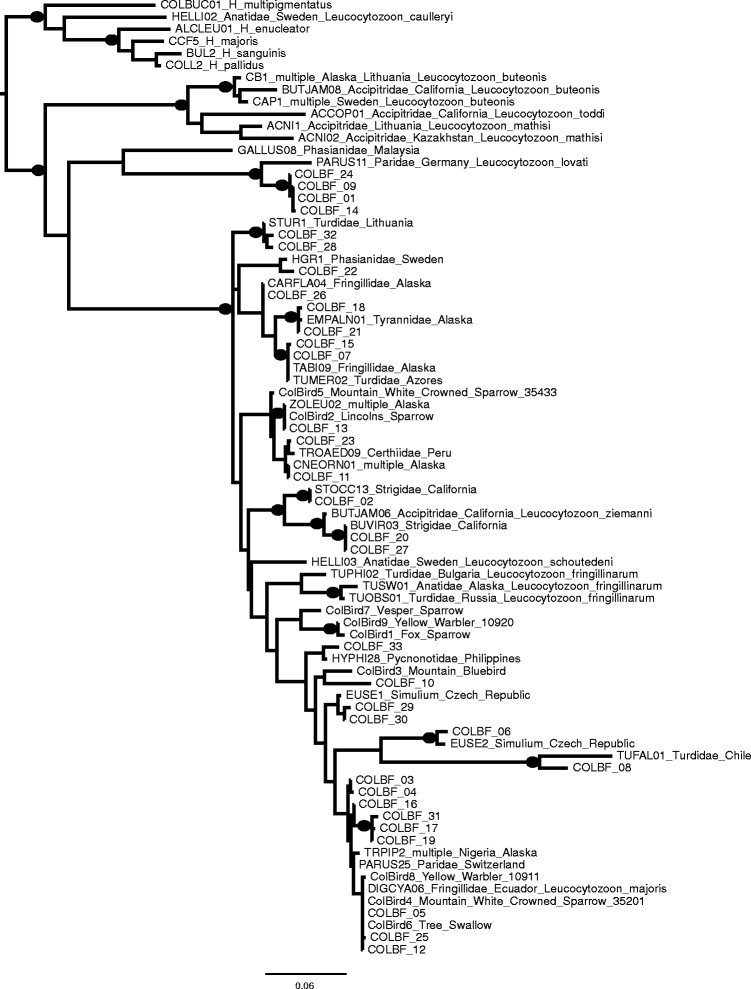
Maximum likelihood phylogeny of *Leucocytozoon cyt b* sequences obtained from Colorado black fly species (indicated as COLBF), their best BLAST match in the MalAvi database, named *Leucocytozoon* species in the database and sequences from *Leucocytozoon* from nine birds (indicated as ColBird) sampled at the same Colorado location. Nodal support values > 95 % are indicated with a black circle on the node

Figure 2 shows a more detailed phylogeny of the 33 *Leucocytozoon* haplotypes that we obtained from the black flies. This phylogeny was analyzed in the context of the nine sequences of *Leucocytozoon* from passerine birds captured at the same site [14]. Also included were the top BLAST matches for each of the black-fly-borne parasites in the MalAvi database and a set of MalAvi haplotypes that came from morphologically identified species of *Leucocytozoon*. Overall, the nodal support of this phylogeny is low, owing to the limited diversity and small fragment size analyzed. However, a few patterns from the analysis are worthy of mention. First, in no case, did any of the black-fly-borne *Leucocytozoon* haplotypes that we obtained exactly match haplotypes detected in birds from the same site; this suggests that the potential vector(s) feed(s) primarily on non-passerines, which comprise the majority of the birds sampled for blood parasites on these field sites. However, one haplotype (COLBF_10) did cluster with the parasites sequenced from a Mountain Bluebird at RMBL, and several of the fly-borne haplotypes (COLBF_11, COLBF_13, and COLBF_23) were part of a clade that included a haplotype isolated from a local Lincoln’s Sparrow, as well as haplotypes detected in both Alaskan and Peruvian birds.

Several of our black fly-borne parasite sequences fell within clades that contained haplotypes of *Leucocytozoon* reported in a recent study that sampled four types of blood parasites in passerine birds over a latitudinal gradient in Alaska [17]. For example, COLBF_26 was extremely similar to haplotype CARFLA04 (Table 1), which was obtained from a Redpoll, a bird that is partially migratory. COLBF_07 and COLBF_15 were similar to a sequence from a parasite in a Two-barred Crossbill (Table 1), another bird that is a resident in both Alaska and Colorado. Although these results could suggest that parasite genotypes move between these locales in either migratory birds or spreading populations, there are also numerous cases where close relatives come from geographically distant locales, such as the clustering of COLBF_33 with a parasite sequence detected in the Philippines or two haplotypes (COLBF_28 and COLBF_32) with their closest relative in our tree being from a parasite in a bird sampled in Lithuania. Overall, these results suggest that some common haplotypes have wide geographic ranges and have turned up in locations where a large amount of sampling for avian haemosporidians has occurred (e.g. Lithuania, Sweden), a pattern that has also been observed for the other genera infecting birds, such as the GRW4 lineage of *Plasmodium relictum* [18].

### Parasite-vector associations

We obtained a large diversity of *Leucocytozoon* haplotypes from *S. silvestre,* and the distribution of parasite haplotypes was not strongly affected by avian host, habitat, or time of season. The large diversity of parasite haplotypes recovered from *S. silvestre* suggests that it is probably an important vector for a multitude of *Leucocytozoon* species on our field sites. The degree of overlap in emergence of *S. silvestre* with bird breeding on these sites, its ornithophilic nature, and its abundance across all habitat types and throughout much of the summer season reinforce the vector potential of this black fly species and might explain why there were no strong effects of avian host, habitat, and time of season on the distribution of *Leucocytozoon* haplotypes. Both *S. silvestre* and *S. craigi* feed on a variety of avian hosts [reviewed in 7]. Based on the sheer number of individuals recovered from light traps, *S. silvestre* was the most abundant ornithophilic black fly on our field sites, and its abundance was not restricted by habitat type. Further, *S. silvestre* reaches peak abundance during the time of season (June 25 – July 14) when most bird species are hatching and feeding susceptible young of the year on our field sites [14]. Other studies have implicated *S. silvestre* as vectors for a number of *Leucocytozoon* species [summarized in 7].

The presence of parasite haplotypes amplified from *G. denaria*, a black fly species that reaches peak abundance early in the summer season [14], further indicates that these parasite haplotypes may come from non-passerine bird hosts we were unable to sample in the field (i.e. resident species or bird species in different orders). Hosts of *Greniera* species are poorly known; the only host record for the entire genus is the Black Grouse (*Tetrao tetrix*) from a study in northern Finland [19]. A wider sampling of the avian and ornithophilic black fly communities is needed to achieve a concrete understanding of how avian hosts, black fly vectors, and environmental factors may shape the distribution of *Leucocytozoon* species on our field sites and globally.

## Conclusions

To our knowledge, this is the first study to amplify parasite DNA from non-engorged, host-seeking black flies. Previous studies on field-captured black flies amplified a diversity of *Leucocytozoon* DNA from blood-fed individuals [6]. However, parasite DNA amplified from the abdomens of blood-fed individuals does not provide proof of vector competence. Not all *Leucocytozoon* species ingested with a bloodmeal can develop infectious stages in the salivary glands of a given black fly species and successfully transmit to the next avian host. These results present only tentative links among *S. silvestre*, a variety of alpine birds, and potential transmission of *Leucocytozoon* parasites. The black flies in our study were collected in CO_2_-baited CDC light traps and were not blood engorged; therefore, most can be presumed to be host-seeking individuals. Thus, parasite sequences amplified from these individuals may potentially be generated from parasite transmission stages, indicating that *S. silvestre* is a competent vector for these *Leucocytozoon* strains. Thus, sampling avian haemosporidian parasites via the collection and screening of host-seeking vectors may provide a means of obtaining a broader sampling of the genetic diversity of these parasites within a locality.

## Additional file

Additional file 1:
**Trapping data for the black fly species captured in Colorado and the number of individuals for each species sampled in 2007 by field site.** Species in bold type are ornithophilic.

## Abbreviations

RMBL: Rocky mountain biological labs
CDC: Centers for disease control
PCR: Polymerase chain reaction
Cytb: Cytochrome oxidase subunit b
