# Supplementary material for: Molecular analyses on host-seeking black flies (Diptera: Simuliidae) reveal a diverse assemblage of Leucocytozoon (Apicomplexa: Haemospororida) parasites in an alpine ecosystem
Source: Parasit Vectors. 2015 Jun 25;8:343. doi: 10.1186/s13071-015-0952-9 (PMC4486084; doi:10.1186/s13071-015-0952-9)
Supplement: Additional file 1: — Trapping data for the black fly species captured in Colorado and the number of individuals for each species sampled in 2007 by field site. Species in bold type are ornithophilic. [file 13071_2015_952_MOESM1_ESM.docx]

**Additional File**

Trapping data for the black fly species captured in Colorado and the number of individuals for each species sampled in 2007 by field site. Species in bold type are ornithophilic.
